# Intimate partner violence and the dual burden of anxiety and depression among women in Zambia: Spatial inequalities and implications for the sustainable development goals

**DOI:** 10.1017/gmh.2026.10261

**Published:** 2026-06-25

**Authors:** Md Salek Miah, Mohammad Shahab Uddin

**Affiliations:** 1 Statistics, Shahjalal University of Science and Technology, Sylhet, Bangladesh; 2 Management, University of Chittagong, Bangladesh; 3 Management, University of Essex Faculty of Social Sciences, UK

**Keywords:** mental health, spatial, IPV, SDGs, LMICs

## Abstract

This study evaluated the association between Intimate Partner Violence (IPV) and mental health indicators, providing evidence to inform maternal health policy. This study used the first nationally representative cross-sectional data on mental health symptoms from the 2024 Zambia Demographic and Health Survey (ZDHS), including 13,951 women aged 15–49 years. Anxiety and depression were assessed using the GAD-7 and PHQ-9 scales, with a score of ≥10 indicating the presence of symptoms. Stepwise, survey-weighted multivariable logistic regression was employed to examine the associations of mental health indicators and various dimensions of IPV, reporting adjusted odds ratios (aORs) and 95% confidence intervals (CIs). Physical IPV was significantly associated with higher odds of Depression aOR= 3.89; 95% CI: 2.16-6.95; p < 0.001), while Emotional IPV was also associated with higher odds of Depression aOR = 3.50; 95% CI: 2.09-5.86; p < 0.001). Similarly, Any IPV was associated with substantial higher odds with Depression aOR = 2.90; 95% CI: 1.80-4.67, p < 0.001) and Anxiety aOR = 2.05; 95% CI: 1.20-3.50, p = 0.008). These findings provide evidence-based, actionable insights for policymakers to integrate mental health and IPV prevention in high-burden provinces to meet the targets of SDG 3 and SDG 5.

## Impact statement

Intimate partner violence (IPV) remains a widespread but under-recognized threat to women’s mental health, particularly in low- and middle-income countries. This study provides nationally representative data in Zambia that indicates that women who experience IPV have significantly increased chances of developing depression and anxiety; some provinces bear a heavier burden than others. Mental health screening instruments combined with spatial analysis reveal geographic disparities that are not evident from national averages alone. The mental health screening instruments, coupled with spatial analysis, show that there are some geographic disparities evident that are not highlighted by national averages. The findings directly relate to the public health planning and realization of the Sustainable Development Goals, namely, SDG 3 (Good Health and Well-being) and SDG 5 (Gender Equality). Identifying high-burden provinces allows policymakers to move beyond broad, mass-based interventions and focus resources on mental health and violence-prevention services where they are most needed. Recognizing high burden provinces enables policymakers to eliminate mass-based solutions and instead direct their efforts toward mental health and violence-prevention services where such needs are the greatest. By incorporating mental health screening and psychosocial support in the current maternal health, primary care and gender-based violence interventions, women at risk may be identified and assisted earlier. Integrating mental health screening and psychosocial support into maternal health and gender-based violence programs can help identify at-risk women early and provide timely support. Outside Zambia, the research provides the experiences of other nations with low- and middle-income populations experiencing the same gender norms, social inequalities and resource limitations. These findings offer insights for other LMICs facing similar challenges, supporting equity-focused interventions. On the whole, this study advocates for more specific, complicated and equity-based measures to minimize the mental impact of intimate partner violence and to promote the health and well-being of women.

## Introduction

Psychological and emotional well-being are termed as mental health (WHO, 2022). Mental health disorders, notably depression and anxiety, constitute one of the most burdensome global public health challenges of the twenty-first century (WHO, 2015). These hardships are expected to affect over 1 billion people worldwide by 2025 and are primary drivers of morbidity, substantially increasing Years Lived with Disability (YLDs) (WHO, 2025). Basically, mental health represents a portion of overall well-being, and disruptions in emotional regulation, cognition or behavior can result in mental disorders that require targeted care and support. Globally, mental disorders considered the leading causes of disease burden, affecting nearly one in eight individuals (Fan et al., 2025). In 2021, ~970 million people were living with mental disorders, including large populations across sub-Saharan Africa (Alam K and GBD 2021 Forecasting Collaborators, 2024). These status highlights substantially to disability and economic loss, with depression and anxiety alone for an estimated US$1 trillion in annual global productivity loss (Brauer et al., 2024).

Furthermore, the burden of common mental disorders is highly unequal. Women are disproportionately affected compared with men, a disparity heavily mediated by social and structural determinants, including pervasive gender-based violence (GBV) (“Global, Regional, and National Burden of 12 Mental Disorders in 204 Countries and Territories, 1990–2019: A Systematic Analysis for the Global Burden of Disease Study 2019”, 2022; C. J. L. Murray et al., 2018). IPV shows a fundamental violation of human rights and a major social determinant of adverse health outcomes among women, encompassing physical, sexual and emotional abuse (Antabe et al., 2025). There is a robustly established causal pathway between exposure to IPV and mental health disorders, consistently shown across varied global contexts (Ali and Zuberi, 2012; Muchemwa et al., 2025; Miah and Kabir, 2026).

One recent, large-scale meta-analysis synthesized evidence demonstrating that survivors of IPV face nearly double the odds of experiencing significant depressive symptoms (Dokkedahl et al., 2022; Flor et al., 2026). As a result, the mental health consequences extend across a scale, including generalized anxiety, post-traumatic stress disorder (PTSD) and diverted suicidal tendency (Dokkedahl et al., 2022). Notably, the psychological and emotional dimensions of IPV are particularly corrosive and often initiate a vicious cycle of eroded self-esteem, diminished self-efficacy and impaired capacity for appropriate care-seeking or exiting an abusive relationship (Babandi et al., 2024; Francis-Tan, 2024). Addressing this IPV-mental health comorbidity is thus an imperative for global health equity (Rasch et al., 2018; Tesfaye et al., 2025). The burden of both IPV and resultant mental health distress is acutely concentrated in LMICs, which bear ~75–85% of the global mental health disease burden (Semrau et al., 2015; Liu et al., 2025). SSA bears a disproportionate share of this burden, with a very high prevalence of IPV coupled with systemic structural inequalities, profoundly constrained mental health services and severe stigma at the community level (Tharp et al., 2013). New national data from countries across the SSA region, such as Mozambique, and earlier waves of surveys in Zambia continue to demonstrate significant associations between IPV exposure and elevated mental health disorder symptoms among women (Antabe et al., 2025).

Mental health problems, especially those of depression and anxiety, are currently one of the biggest challenges worldwide, and the problem mostly affects women (Lund et al., 2018). In the case of Zambia, the large scale of IPV, combined with the shortcomings of mental health interventions and various inequalities, makes it imperative to recognize the mental health problems for effective interventions and policy formulations (Patel et al., 2018).

Specifically, Zambia has long reported among the highest rates of IPV for women aged 15–49 years in the region (Musaka and Musekiwa, 2024). Such violence is usually sustained by deep-seated and pervasive gender norms that afford men authority and sometimes justify violence against women (Afifi, 2007). First, the older nationally representative surveys, such as the ZDHS 2013–2014, often lacked standardized and validated screening scales needed to capture common mental disorders like anxiety and depression, together with comprehensive data on IPV exposure (Winter et al., 2021; Jia et al., 2025). In the absence of spatially disaggregated evidence, policy responses will fall to an inefficient one-size-fits-all national approach, overlooking critical regional differences and possibly failing to reach the most vulnerable populations with sufficient resources (AL-shahrani and Hammad, 2025). Besides, some South Asian countries, including Nepal and Bangladesh, conducted these types of investigations to identify the key risk factors of anxiety and depression (Pandey et al., 2024; Miah & Ullah, 2025a). Additionally, Mozambique also performed similar types of study, but there are socioeconomical, cultural and geographical gaps arising (Zhang et al., 2019).

While various research studies conducted in various countries, Nepal, Mozambique, Lesotho, and Bangladesh, for instance, had explored the connections between intimate partner violence and mental health outcomes, none of them had conducted the study on the national representative level in the case of ZDHS, which had various shortcomings when considering spatially and standardized mental health indicators (Munakampe, 2020; Pandey et al., 2024; Rahman et al., 2024; Melkam et al., 2025; Miah & Ullah, 2025a; Miah and Kabir, 2026). The 2024 ZDHS, which, for the first time, includes the validated Patient Health Questionnaire-9 (PHQ-9) and Generalized Anxiety Disorder-7 (GAD-7) sections, makes it possible to overcome the gaps and provide the first comprehensive information regarding the situation of anxiety and depression, as well as intimate partner violence, in the case of Zambian women (Jacobs et al., 2017).

Therefore, to address this evidence and geographical gap, the researchers emerged with the key insights of mental health symptoms to inform the policymakers. Therefore, this study robustly addresses this gap using necessary spatial and recent data, leveraging the update released and comprehensive 2024 Zambia Demographic and Health Survey (ZDHS).

The primary research question is what is the impact of intimate partner violence on the mental health of women in Zambia, and the differences in the prevalence of anxiety and depression in various regions? An additional research question is how the prevalence and associations of intimate partner violence and mental health outcomes (anxiety and depression) vary across different provinces in Zambia, highlighting spatial inequalities in mental health burden?

The robust evidence addressed by this research is strongly aligned with global and national health policies, most notably the Sustainable Development Goals (SDGs) – specifically, SDG 3 (Good Health and Well-being, target 3.4 for mental health), SDG 5 (Gender Equality, target 5.2 to eliminate violence against women) and SDG 10 (Reduced Inequalities).

Therefore, this study aims to:

1. Assess the associations between all dimensions of IPV (physical, sexual, emotional and any IPV) and mental health indicators (anxiety and depression) among women aged 15–49 years in Zambia using the 2024 ZDHS data.
2. Examine and map the subnational spatial variation and heterogeneity of this mental health and IPV prevalence across Zambia’s provinces, providing geographically focused evidence for policy-making.

This study provides novel, nationally representative evidence on anxiety and depression among Zambian women using the 2024 ZDHS, the first survey to include validated mental health modules (PHQ-9 and GAD-7). It offers policy-relevant spatial insights by mapping subnational inequalities in mental health and various dimensions of IPV prevalence, highlights nuanced associations across all IPV dimensions and reinforces evidence for integrated mental health and IPV interventions. The findings support improvement to SDGs 3, 5 and 10 and generate convenient information for other LMICs with limited mental health data, informing targeted policies and programs.

This study follows the subsequent order: Section 2 presents the methodology, including study design, data, variables, analyses and ethical considerations. Section 3 reports the results, summarizing participant characteristics, prevalence, associations and spatial patterns. Finally, Section 4 provides the discussion, highlighting key findings, interpretation, implications, limitations and recommendations for future research.

## Methodology

### Data source

This study is based on the nationally representative cross-sectional dataset of the most recent seventh round 2023–2024 ZDHS. The survey was conducted by the Zambia Statistics Agency (*ZamStats*) in collaboration with the Ministry of Health (MoH), the University Teaching Hospital Virology Laboratory (UTH-VL), the Tropical Diseases Research Centre (TDRC) and the University of Zambia (UNZA). ICF International, under the DHS program, provided the additional assistance, and the survey was conducted from 17 January to 7 July 2024 using computer-assisted personal interviews (CAPIs). Funding and support were provided by the Government of Zambia, the United States Agency for International Development (USAID), the Global Fund to Fight AIDS, Tuberculosis and Malaria and UNICEF (Kamanga et al., 2022; Musaka and Musekiwa, 2024).

### Sampling design, setting and sample size

The ZDHS 2023–2024 is a nationally representative survey that used a two-stage stratified random sampling design. A complete list of enumeration areas (EA) covering the whole country was prepared by *ZamStats* based on the most recent population (seventh round) and housing census, and it was used as the sampling frame. The primary sampling unit (PSU) for the survey is an EA, which contains ~130–150 households on average. In total, 14,000 women aged 15–49 years were eligible for individual interviews, of whom 13,951 women completed the interviews, yielding a response rate of 99.6%. For this study, only women who completed the mental health module (assessing anxiety via GAD-7 and depression via PHQ-9) were included. Consequently, the analysis is based on a sample of 13,951 women (Supplementary Figure S1). No patients are included in this study. For further details on the sampling procedure, data collection process, quality control measures and the questionnaires developed for this survey, namely, the household questionnaire, individual women’s questionnaire and domestic violence module, the readers are referred to the ZDHS 2023–2024 report (Musaka and Musekiwa, 2024). This study maintained all items of Strengthening the Reporting of Observational Studies in Epidemiology (STROBE) guidelines (Cuschieri, 2019).

### Variables

### Outcome variables

Anxiety and depression were the main outcomes of this study. The anxiety indicator was assessed using the 7-item Generalized Anxiety Disorder Scale (GAD-7), a self-reported questionnaire that measures the presence and severity of generalized anxiety symptoms over the past 2 weeks. Participants rated each item on a four-point Likert scale starting from 0 (never) to 3 (always), yielding total scores between 0 and 21 (Casares et al., 2024). The second outcome variable, the depression indicator, was measured using the 9-item Patient Health Questionnaire (PHQ-9), which assesses depressive indicators over the preceding 2 weeks. Responses were also recorded on a four-point Likert scale, yielding total scores from 0 to 27. Severity was classified as follows: 0–4 (no depression), 5–9 (mild), 10–14 (moderate), 15–19 (moderately severe) and 20–27 (severe), with scores of ≥10 indicating moderate to severe depression (Manea et al., 2012). For this study, both depression and anxiety were dichotomized (1 = yes, 0 = no) using established cutoff points (PHQ-9 ≥ 10 and GAD-7 ≥ 10), consistent with prior literature. The PHQ-9 and GAD-7 demonstrated strong internal consistency, with Cronbach’s *α* of 0.821 and 0.834, respectively (Steinman et al., 2025; Lushington et al., 2026; Yi et al., 2026).

To address potential co-occurring symptoms, anxiety and depression were analyzed independently; however, all multivariable models were mutually adjusted, meaning depression was included as a covariate in anxiety models and vice versa. This approach reduces bias arising from overlapping symptomatology.

Additionally, respondents who reported receiving treatment (medication or counseling) but scored below the clinical thresholds were retained and classified as “0 = no symptoms,” following DHS psychometric guidelines, since the objective was to estimate current symptom burden rather than treatment history (Pengpid et al., 2025; Steinman et al., 2025; Miah & Ullah, 2025a.

### Exposures variables

#### Intimate partner violence (IPV)

The primary exposure to IPV was classified as physical, emotional, sexual and any IPV, following WHO and DHS definitions (Table 1):

- Physical IPV indicated acts such as being pushed, shaken, slapped, punched, kicked, strangled, attacked with a weapon or having the arm twisted/pulled (d105a–d105f, d105j).
- Emotional IPV indicated humiliation, threats or insults (d103a–d103c).
- Sexual IPV indicated forced intercourse or other unwanted sexual acts (d105h–d105k).
- Any IPV captured exposure to at least one form.

**Table 1.** Classification of IPV exposures Table 1. long description.

Exposure	Definition	Examples/acts	Variables (DHS codes)	Coding
Physical IPV	Physical aggression by a partner	Pushed, shaken, slapped, punched, kicked, strangled, attacked with a weapon, arm twisted/hair pulled	d105a: pushed/shaken/object thrown d105b: slapped d105c: punched/hit by something harmful d105d: kicked, dragged, beaten d105e: choked or burned d105f: threatened with knife/gun/weapon d105j: arm twisted/hair pulled	1 = experienced any of the above; otherwise, 0 = none
Emotional IPV	Emotional harm or fear	Humiliation, threats, insults	d103a: humiliated, d103b: threatened with harm, d103c: insulted or made to feel bad	1 = experienced any of the above, 0 = none
Sexual IPV	Sexual act forced by a partner	Forced intercourse or unwanted sexual acts	d105h: physically forced into unwanted sex, d105i: forced into other unwanted sexual acts, d105k: forced with threats/other means to perform unwanted acts	1 = experienced any of the above, 0 = none
Any IPV (composite)	Exposure to at least one type of IPV	Physical, emotional or sexual IPV	Physical, emotional, sexual (see above)	1 = exposure to any IPV, 0 = none

For this study, all IPV exposures were classified as a binary category (1 = yes and 0 = no).

Importantly, exposure to one form of IPV did *not* preclude exposure to the others. Women could experience physical, emotional and sexual IPV simultaneously, and these categories were treated as non-mutually exclusive, consistent with DHS methodology.

### Covariates

In this study, socioeconomic and demographic features of women, such as their age in years,(categorized into 15–24, 24–25 and 35–49), age at first birth in years (10–17, 18–24 and ≥ 25), age at cohabitation in years (<18, 18–21 and > 21), women education level (no education, primary and secondary + higher), number of children (no children, 1–2 children and 3+ children), maternal working status (not working and currently working), household wealth status (poor, middle class and rich), place of residence (rural and urban), household religion status (Non-Protestant and Protestant), number of the household members (<5 and ≥ 5), household ownership (does not own and owns), contraception decision status (not joint and joint), household media access (no media and has media), use of internet status (no and yes), household assets (no major asset and yes) and household materials (unimproved and improved). Additional household-level variables were constructed to capture specific living conditions. Household ownership was recoded as a binary variable (owns vs. does not own). A composite household asset variable was generated, coded as “yes” if the household owned at least one major asset (refrigerator, bicycle, motorcycle/scooter or car/truck). Household materials were also categorized as improved versus unimproved based on wall, floor and roof materials, following standard DHS classifications. These variables were included alongside the DHS wealth index to distinguish overall socioeconomic status from specific material and environmental conditions (Vyas and Kumaranayake, 2006; Thomson et al., 2009; Howe et al., 2012). This selection of covariates in this study was used by several factors, including their availability in the ZDHS 2023–2024 dataset, relevant studies and self-consideration (Anik et al., 2021; Mahfuzur et al., 2022; Hasan et al., 2023; Shawon et al., 2024; Negash and Wubneh, 2025; Pengpid et al., 2025; Rafi et al., 2025; Miah and Ullah, 2025b; Miah et al., 2026).

### Confounder selection

This portion provides the methods applied to detect and select potential confounders for adjustment in this study. The confounder selection was based on statistical testing and conceptual analysis to ensure the robustness and validity of the final adjusted model.

#### Step 1: Bivariate analysis

Initially, this study used chi-square tests to assess associations between covariates and mental health indicators. Categorical variables with *p* < 0.25 were retained as potential confounders for adjusting final multivariate modeling to avoid excluding potentially important determinants (Celentano and Szklo, 2019).

#### Step 2: Forward stepwise model selection

In this stage, all potential confounders as covariates were included one by one in the model based on statistical (*p* < 0.25) and conceptual considerations. At each step, the inclusion of a variable was evaluated based on:

- Statistical significance (*p*-value).
- The changes in odds ratio (OR) for the main exposure variable.
- Model fit statistics, Akaike Information Criterion (AIC) and Bayesian Information Criterion (BIC).

Conceptually aligned confounders were retained even if they slightly increased AIC/BIC, preventing model misspecification. Separate models were fitted for depression and anxiety, including IPV as the main exposure. Additionally, this study used likelihood ratio tests (LRT) to compare nested logistic regression models, evaluating the effect of covariates on model performances. The statistical significance of the LR test (*p*-value) showed that the full model, including demographic and household variables, provided a significantly better fit than the reduced model. In addition, model fit was further evaluated using the Akaike Information Criterion (AIC) and Bayesian Information Criterion (BIC).

### Bias and missing handling

Participants with missing information on PHQ-9 and GAD-7, or IPV exposures were excluded from the analysis (complete-case analysis). Additionally, survey weights, clustering and stratification were applied according to ZDHS guidelines to reduce bias. Weighting provides that the survey estimates are nationally representative and adjust for unequal probabilities of selection and nonresponse.

### Model assumption

After developing the final adjusted model, the *Hosmer-Lemeshow* goodness-of-fit test was applied to assess the model’s fit; a nonsignificant *p*-value indicated a good fit. Additionally, for evaluating multicollinearity, variance inflation factors (VIFs) were used, and a VIF value >10 shows potential concerns with collinearity in covariates.

### Set of confounders adjusted in final model

This following set of confounders are added in final adjusted stepwise survey-weighted multivariable logistic regression model, including maternal age, maternal education, household wealth status, place of residence, maternal employment, number of living children, age at cohabitation, household head sex, media access, internet status, household materials, household assets, abstaining status, menstruation status, administrative province, contraception status, religion status, household size, household ownership and health card access.

### Statistical analysis

### Descriptive analysis

In this investigation, descriptive analysis was used to summarize and to explore the background characteristics of the study populations, which were presented with the frequency and percentage for all categorical variables. Additionally, several donut charts were visualized for showing prevalences of anxiety, depression and various dimensions of IPV prevalence.

### Bivariate analysis

This study used Pearson’s *χ* ^2^-test to assess the initial significant association between study variables and mental health indicators in ever-married women aged 15–49 years, and the results were presented as cell percentages for all categorical cross-tabulations.

### Multivariate analysis

**Model 1**: Unadjusted/crude model (IPV exposure only).

**Model 2**: Adjusted for demographic variables as potential confounders conceptually and statistically (age, education, religion, province, etc.).

Finally, in this process, a stepwise survey-weighted multivariable logistic regression model was applied to assess the association between mental health indicators (anxiety and depression) and IPV exposure. All results were presented as 95% confidence interval (CI) and adjusted odds ratios (aORs).

### Spatial analysis

Additionally, this study used several choropleth plots to assess the spatial variation of mental health indicators and IPV prevalence across provinces. Initially, prevalence statistics for mental health indicators and IPV were calculated in Stata and exported in MS Excel format. The Excel datasets were subsequently imported into R (version 4.5.1), and the datasets were merged with Zambian administrative boundary shapefiles (https://data.humdata.org/dataset). Finally, spatial data preprocessing and geospatial visualization were applied using the *sf*, *tidyverse*, *ggplot2* and *tmap* packages.

### Analytical tools

All analyses were performed using statistical software STATA version 17 (StataCorp LLC, College Station, TX, USA) and R version 4.5.1 with R Studio (Posit PBC, Boston, MA, USA).

## Results

### Background characteristics

Among all women, about 1.3% of individuals reported anxiety, and 1.6% reported depression (Supplementary Figure S2). Women exposed to any IPV showed a significantly higher proportion of depression (11.59% among depressed women) and anxiety (10.71% among anxious women) compared to women not exposed to IPV (*p* < 0.001 for both outcomes). Among IPV subtypes, physical IPV and emotional IPV were significantly associated with both depression and anxiety. Physical IPV was more common among women with depression (7.73%) and anxiety (5.36%), while emotional IPV showed stronger associations with depression (9.66%) and anxiety (7.14%), and both were statistically significant (*p* < 0.001). Sexual IPV showed a statistically significant association with anxiety (2.98%, *p* = 0.014). Among sociodemographic factors, maternal age showed a significant association with both outcomes (*p* ≤ 0.001), with a higher proportion of depression and anxiety observed among older women aged 35–49 years. Maternal education was significantly associated with depression (*p* = 0.041), while household wealth was also associated with depression (*p* = 0.005). Residence type showed significant associations with both depression and anxiety, with higher proportions observed in urban areas. Access to household media and internet use were significantly associated with both outcomes. Women reporting internet use had a higher proport*i*on of depression (2.12%) and anxiety (1.70%) compared to nonusers (*p* < 0.001 and *p* = 0.002, respectively). Household material quality was significantly associated with both depression and anxiety (*p* = 0.008 and *p* = 0.010, respectively), indicating variation in mental health outcomes across living conditions. However, variables such as age at first birth, age at cohabitation, pregnancy status, household size and household ownership were not significantly associated with either depression or anxiety (Table 2).

**Table 2.** Bivariate association between intimate partner violence, sociodemographic characteristics and maternal mental health outcomes (depression and anxiety) among women in Zambia (*N* = 13,951) Table 2. long description.

Characteristics	Depression (PHQ-9 ≥ 10)			Anxiety (GAD-7 ≥ 10)		
	No (%)	Yes (%)	*p*	No (%)	Yes (*n*%)	*p*
**IPV exposures**						
**Physical IPV**			**<0.001**			0.009
No	13,433 (97.74)	311(2.26)		13,465 (97.69)	318 (2.31)	
Yes	191 (92.27)	16 (7.73)		159(94.64)	9 (5.36)	
**Emotional IPV**			**<0.001**			0.002
No	13,344 (97.09)	400 (2.91)		13,375 (97.04)	408 (2.96)	
Yes	187 (90.34)	20 (9.66)		156 (92.86)	12 (7.14)	
**Sexual IPV**			0.212			0.014
No	13,601 (98.96)	143 (1.04)		13,641 (98.97)	141 (1,03)	
Yes	203 (98.07)	4 (1.93)		16,397.02)	5 (2.98)	
**Any IPV**			**<0.001**			**<0.001**
No	13,132 (95.55)	612 (4.45)		13,165 (95.52)	618 (4.48)	
Yes	183 (88.41)	24 (11.59)		150 (89.29)	18 (10.71)	
**Sociodemographic characteristics**						
**Maternal age**			0.001			**<0.001**
15–24 years	5,826 (98.93)	63 (1.07)		5,843 (99.22)	46 (0.78)	
25–34 years	4,049(98.42)	65 (1.58)		4,054 (98.54)	60 (1.46)	
35–49 years	3,869 (98.00)	79 (2.00)		3,886 (98.43)	62 (1.57)	
**Age at first birth**			0.250			0.712
10–17 years (Early)	3,958 (98.26)	70 (1.74)		3,976 (98.71)	52 (1.29)	
18–24 years (Normal)	5,484 (98.56)	80 (1.44)		5,502 (98.89)	62 (1.11)	
≥25 years (Late)	4,299 (98.69)	57 (1.31)		4,302 (98.76)	54 (1.24)	
**Age at cohabitation**			0.757			0.550
<18 years (Young)	3,901 (98.63)	54 (1.37)		3,905 (98.74)	50 (1.26)	
18–20 years (Mild)	2,836 (98.44)	45 (1.56)		2,852 (98.99)	29 (1.01)	
≥21 years (Older)	7,007 (98.48)	108 (1.52)		7,026 (98.75)	89 (1.25)	
**Maternal education**			0.041			0.222
No education	947 (98.75)	12 (1.25)		950 (99.06)	9 (0.94)	
Primary	5,778 (98.79)	71 (1.21)		5,787 (98.94)	62 (1.06)	
Secondary or higher	7,019 (98.26)	124 (1.74)		7,046 (98.64)	97 (1.36)	
**Number of living children**			0.109			0.062
No children	3,776 (98.80)	46 (1.20)		3,781 (98.93)	41 (1.07)	
Few (1–2)	4,319 (98.58)	62 (1.42)		4,338 (99.02)	43 (0.98)	
Many (3+)	5,649 (98.28)	99 (1.72)		5,664 (98.54)	84 (1.46)	
**Maternal employment**			0.239			0.136
Not working	7,338 (98.63)	102 (1.37)		7,360 (98.92)	80 (1.08)	
Currently working	6,406 (98.39)	105 (1.61)		6,423 (98.65)	88 (1.35)	
**Currently pregnant**			0.975			0.889
No	12,807 (98.52)	193 (1.48)		12,843 (98.79)	157 (1.21)	
Yes	937 (98.53)	14 (1.47)		940 (98.84)	11 (1.16)	
**Number of unions**			0.004			0.251
Once	7,714 (98.61)	109 (1.39)		7,731 (98.82)	92 (1.18)	
More than once	1,464 (97.60)	36 (2.40)		1,477 (98.47)	23 (1.53)	
**Currently abstaining**			0.673			0.005
No	12,722 (98.53)	190 (1.47)		12,766 (98.87)	146 (1.13)	
Yes	1,022 (98.36)	17 (1.64)		1,017 (97.88)	22 (2.12)	
**Menstruated in last 6 Weeks**			0.111			0.154
No	4,206 (98.27)	74 (1.73)		4,220 (98.60)	60 (1.40)	
Yes	9,538 (98.62)	133 (1.38)		9,563 (98.88)	108 (1.12)	
**Household head Sex**			0.411			0.244
Male	9,333 (98.57)	135 (1.43)		9,361 (98.87)	107 (1.13)	
Female	4,411 (98.39)	72 (1.61)		4,422 (98.64)	61 (1.36)	
**Household wealth**			0.005			0.145
Low	5,519 (98.89)	62 (1.11)		5,522 (98.94)	59 (1.06)	
Middle	2,797 (98.52)	42 (1.48)		2,809 (98.94)	30 (1.06)	
High	5,428 (98.14)	103 (1.86)		5,452 (98.57)	79 (1.43)	
**Residence type**			0.016			0.007
Urban	6,023 (98.24)	108 (1.76)		6,040 (98.52)	91 (1.48)	
Rural	7,721 (98.73)	99 (1.27)		7,743 (99.02)	77 (0.98)	
**Household religion**			0.400			0.068
Non-Protestant	2,083 (98.72)	27 (1.28)		2,093 (99.19)	17 (0.81)	
Protestant	11,661 (98.48)	180 (1.52)		11,690 (98.72)	151 (1.28)	
**Household size**			0.209			0.277
<5	4,287 (98.33)	73 (1.67)		4,301 (98.65)	59 (1.35)	
5 or more	9,457 (98.60)	134 (1.40)		9,482 (98.86)	109 (1.14)	
**Household ownership**			0.799			0.551
Does not own	8,446 (98.50)	129 (1.50)		8,468 (98.75)	107 (1.25)	
Owns	5,298 (98.55)	78 (1.45)		5,315 (98.87)	61 (1.13)	
**Contraception decision-making**			0.327			0.061
Not joint	10,350 (98.46)	162 (1.54)		10,375 (98.70)	137 (1.30)	
Joint	3,394 (98.69)	45 (1.31)		3,408 (99.10)	31 (0.90)	
**Household media access**			0.006			0.311
No media	3,165 (99.03)	31 (0.97)		3,163 (98.97)	33 (1.03)	
Has media	10,579 (98.36)	176 (1.64)		10,620 (98.74)	135 (1.26)	
**Internet use**			**<0.001**			0.002
No internet	10,287 (98.73)	132 (1.27)		10,311 (98.96)	108 (1.04)	
Internet use	3,457 (97.88)	75 (2.12)		3,472 (98.30)	60 (1.70)	
**Household assets**			0.730			0.759
No major asset	6,141 (98.56)	90 (1.44)		6,154 (98.76)	77 (1.24)	
Owns major asset	7,603 (98.48)	117 (1.52)		7,629 (98.82)	91 (1.18)	
**Household materials**			0.008			0.010
Unimproved	3,571 (98.97)	37 (1.03)		3,579 (99.20)	29 (0.80)	
Improved	10,173 (98.36)	170 (1.64)		10,204 (98.66)	139 (1.34)	

*Note*: Bold values are used to highlight the statistically significant *p*-values. Values are presented as row percentages unless otherwise stated.

### Multivariable analysis of the association between intimate partner violence and mental health outcomes

Women experiencing Physical IPV showed about fourfold higher odds of depression compared to unexposed women (aOR = 3.89; 95% CI: 2.16–6.95, *p* < 0.001). Similarly, Emotional IPV was associated with 3.5-fold higher odds (aOR = 3.50; 95% CI: 2.09–5.86, *p* < 0.001). Overall exposure to Any IPV also significantly increased the odds of depression (aOR = 2.90; 95% CI: 1.80–4.67, *p* < 0.001). Additionally, Emotional IPV and Any IPV were significantly associated with higher odds of anxiety. Women exposed to Emotional IPV had nearly double the odds of anxiety (aOR = 1.97; 95% CI: 1.05–3.72; *p* = 0.034), while exposure to Any IPV doubled the odds of anxiety (aOR = 2.05; 95% CI: 1.20–3.50; *p* = 0.008) (Table 3).

**Table 3.** Associations of IPV exposures and mental health indicators (*N* = 13, 951) Table 3. long description.

Outcome	Exposure	Unadjusted OR (95% CI), *p*-value	Adjusted OR (95% CI), *p*-value
**Anxiety**	Physical IPV (Yes)	1.84 (0.91–3.69), 0.085	1.84(0.89–3.83), 1.84
	Ref (No)	–	–
	Sexual IPV (Yes)	2.50 (0.95–6.59), 0.065	2.46 (0.90–6.70), 0.077
	Ref (No)	–	–
	Emotional IPV (Yes)	2.11 (1.15–3.89), 0.015	1.97(1.05–3.72), 0.034
	Ref (No)	–	–
	Any IPV (Yes)	2.06 (1.23–3.44), 0.005	2.05 (1.20–3.50), 0.008
	Ref (No)	–	–
**Depression**	Physical IPV (Yes)	3.32 (1.93–5.69), **<0.001**	3.89 (2.16–6.95), **<0.001**
	Ref (No)		
	Sexual IPV (Yes)	1.64 (0.58–4.67), 0.53	1.75 (0.60–5.06), 0.298
	Ref (No)		
	Emotional IPV (Yes)	3.29 (2.01–5.40), **<0.001**	3.50 (2.09–5.86), **<0.001**
	Ref (No)	–	–
	Any IPV (Yes)	2.59 (1.64–4.07), **<0.001**	2.90(1.80–4.67), **<0.001**
	Ref (No)	–	–

*Note*: Bold values are used to highlight the statistically significant *p*-values. Reference category (Ref): No These confounders are added in final adjusted model: maternal age, maternal education, household wealth status, place of residence, maternal employment, number of living children, age at cohabitation, household head sex, media access, internet status, household materials, household assets, abstaining status, menstruation status, administrative province(division), contraception status, religion status, household size, household ownership and health card access.

### Spatial distribution of anxiety and depression across Zambia

**Figure 1.**
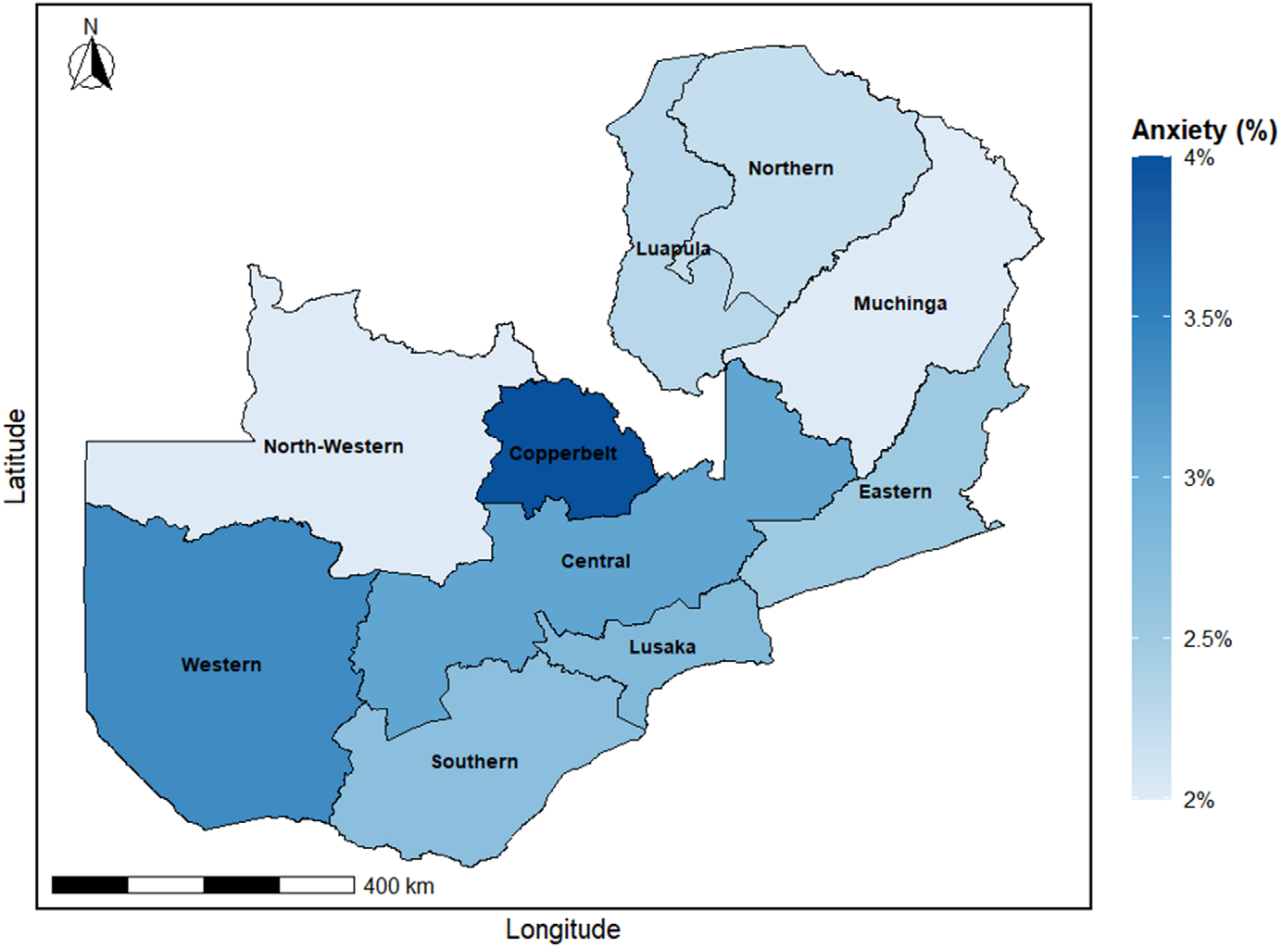
Spatial inequalities of anxiety. Figure 1. long description.

**Figure 2.**
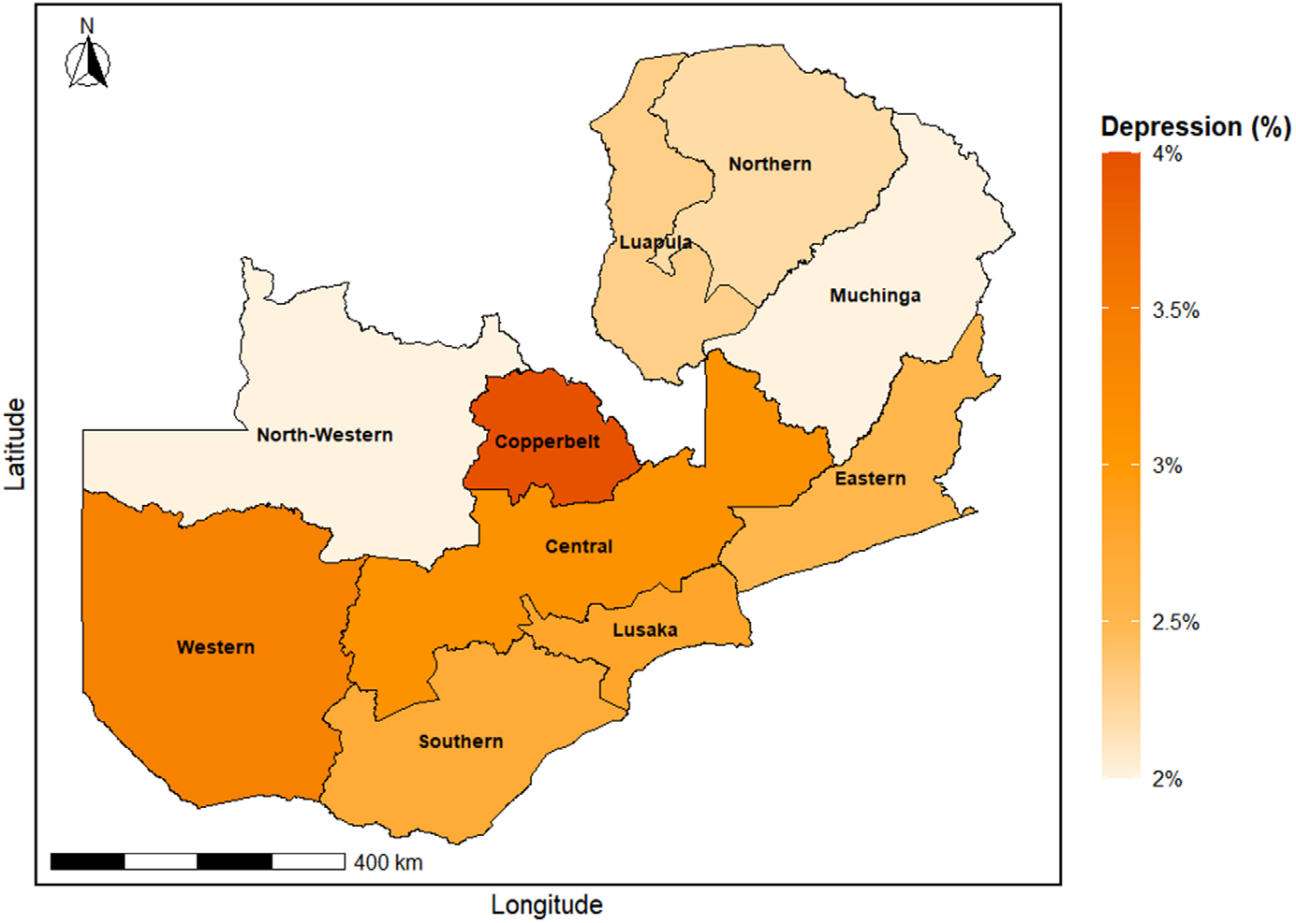
Spatial inequalities of depression. Figure 2. long description.

The highest prevalence of anxiety and depression was shown in the Copperbelt. Besides, moderate levels of anxiety and depression are shown in Central, Eastern, Lusaka and Southern provinces. On the other hand, Northern, Muchinga, Luapula and North-Western provinces showed the lowest prevalence (Figures 1 and 2).

### Spatial distribution of IPV exposures across Zambia

**Figure 3.**
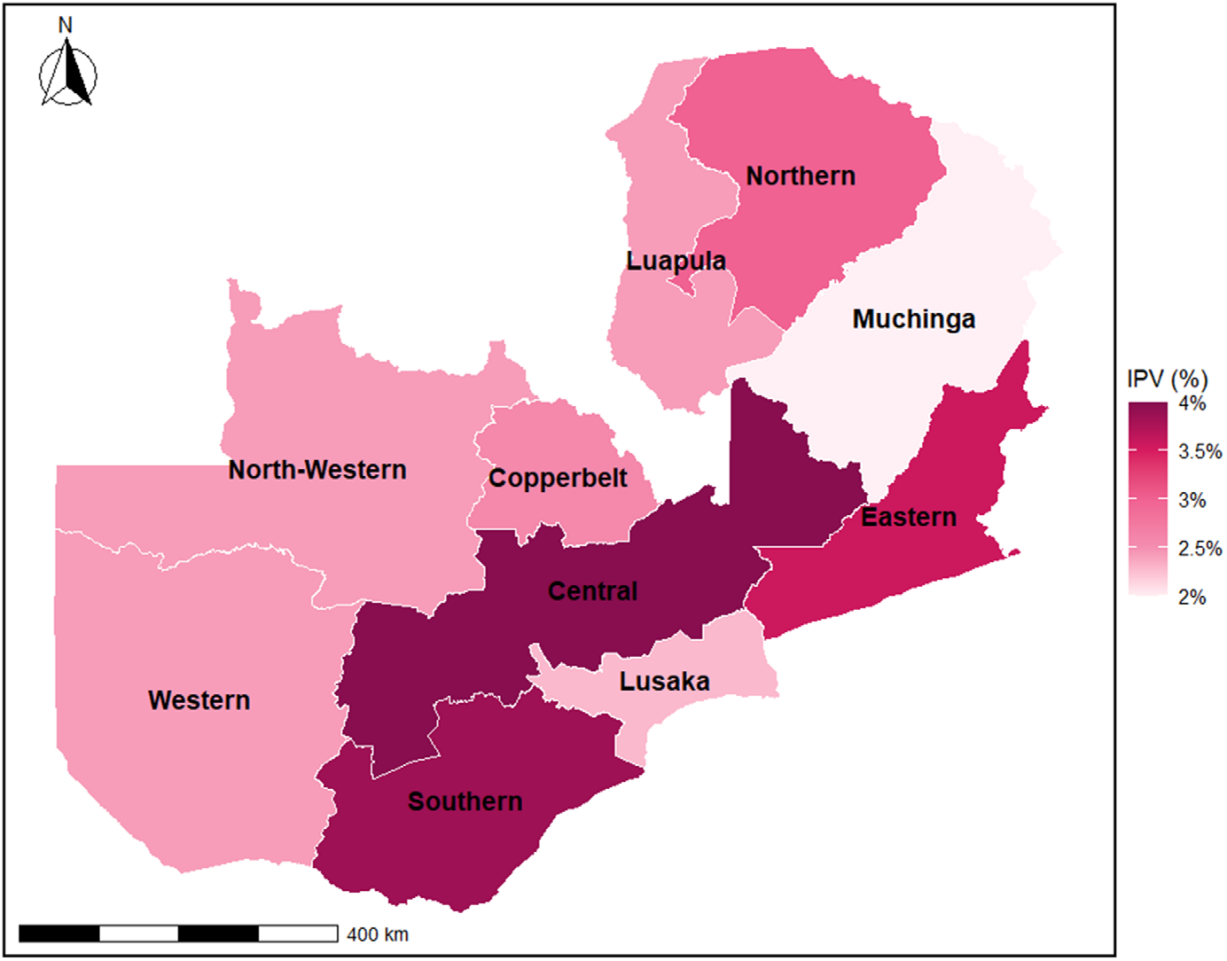
Spatial inequalities of any form of IPV. Figure 3. long description.

The maps reveal substantial geographic variation in all types of intimate partner violence (IPV) across Zambia. Central, Southern and Eastern provinces consistently show the highest IPV burden, while Muchinga, Luapula, Western, Northern and North-Western provinces report comparatively lower prevalence. Central and Southern provinces exhibit the highest kinds of IPV. Emotional IPV showed the highest prevalence in Southern and Central provinces, and lower prevalence showed Northern, North-Western, Muchinga and Luapula. The highest physical IPV was shown in Southern and Central provinces, and Western, Muchinga and Luapula showed the lowest levels. Additionally, the highest Sexual IPV prevalence is in Central province, with notable burdens in Eastern and Southern provinces; Muchinga, Luapula and North-Western provinces report the lowest prevalence. Overall, the spatial variation shows a southern central belt of Zambia as the main location for all forms of IPV (Figure 3 and Supplementary Figures S3–S5).

## Discussion

This national representative sample of 13,951 ever-married Zambian women aged 15–49 years indicates a strong and significant association between intimate partner violence (IPV) and mental health indicators. The odds of depression were almost four times higher among women who were exposed to physical IPV (aOR = 3.89; 95% CI: 2.16–6.95, *p* < 0.001) and 3.5 times higher among women who were exposed to emotional IPV (aOR = 3.50; 95% CI: 2.09–5.86, *p* < 0.001). Exposure to any IPV was associated with an almost threefold risk of depression (aOR = 2.90; 95% CI: 1.80–4.67, *p* < 0.001). Both emotional and any IPV were also strongly associated with anxiety, and the proportion of affected women doubled (Emotional IPV: aOR = 1.97; 95% CI: 1.05–3.72, *p* = 0.034; Any IPV: aOR = 2.05; 95% CI: 1.20–3.50, *p* = 0.008).

The burden of anxiety and depression was shown to be the highest in the Copperbelt province, showing moderate prevalence in Central, Eastern, Lusaka and Southern provinces, and minimal prevalence in Northern, Muchinga, Luapula and North-Western provinces. Equally, the IPV mapping revealed that the burden is continuously recorded to be in the Central, Southern and Eastern provinces, which led to the demonstration of a southern-central focus of vulnerability. Spatial disparities in IPV and mental health highlight the importance of targeted, location-specific mitigation strategies.

The results are largely in line with the evidence in sub-Saharan Africa (Antabe et al., 2025). Research in Zambia, Malawi and Tanzania has reported an increased psychiatric morbidity in women who have been physically and emotionally subjected to IPV, specifically in depression (Brar et al., 2017; Patwardhan et al., 2024; Casella et al., 2025). The current research is based on new evidence with national representative data on standardized instruments of mental health (PHQ-9 and GAD-7) in the 2023–2024 ZDHS dataset, providing both individual-level association and spatial clues (Maila et al., 2022; Mabena et al., 2025). The insignificant relationship between sexual IPV and mental health is in contrast with certain SSA research with high risk (Jacobs et al., 2017). This discrepancy may reflect cultural norms, reporting bias or methodological factors, underlining the need for sensitive measurement and qualitative investigation of sexual IPV in Zambia (Munne et al., 2007; Alam et al., 2024; Tohan et al., 2025).

The prevalence rate for mental health disorders among Zambian women appears low, with 1.3% for anxiety and 1.6% for depression. Though prevalences are low, these represent a meaningful population affected by mental health disorders, potentially due to lack of awareness or stigma, emphasizing the continued importance of mental health screenings (Graham et al., 2024). The findings also emphasize that these could be affected populations that have resulted due to a lack of awareness or stigma associated with these disorders. The findings also endorse the continued importance associated with mental health screenings (Nabila Ashraf et al., 2021; Dadras, 2025).

Spatial analysis shows Southern and Central provinces, along with Eastern province, bear the highest burden of IPV and associated mental health outcomes. On the contrary, Northern, North Western, Muchinga, Luapula and Western provinces had lower IPV prevalence. These observations point toward a southern-central belt region with maximum prevalence of all types of IPV as the major hot spot region, which needs IMCI intervention at the level of this region in view of the prevailing inequality with regard to IPV exposure (Wainberg et al., 2017; Jester et al., 2023). This highlights a southern-central belt hotspot requiring targeted interventions to reduce inequalities in IPV exposure. These regional inequalities may be driven by variations in socioeconomic conditions, cultural norms regarding gender and violence, urbanization and access to reporting mechanisms and health services, which can influence both exposure to IPV and the recognition of mental health needs (Budge et al., 2025; Mokwena and Makhozonke, 2024; S. M. Murray et al., 2025; Shimels et al., 2025; Ullman et al., 2025).

The results revealed that both emotional and physical IPV have significant associations with increased rates of anxiety and depression among Zambian women, though the Sexual IPV had nonsignificant results, possibly due to the culture and stigma associated with it (N. Ahmed et al., 2025; S. Ahmed et al., 2019). While emotional IPV could have widespread psychosocial consequences by causing a reduction in self-esteem and coping mechanisms, it could also be a chronic cause of stress that could further increase the risks associated with both anxiety and depression (Khan, 1985; Sultana et al., 2025). Physical IPV could be a direct cause of trauma, fear and helplessness that could further increase the risks associated with depression (Padgett, 2020; Sen et al., 2023). Exposure to multiple IPV types compounds mental health risks(Zafri et al., 2021; Faruk et al., 2023). The disparities also point toward the fact that women living in provinces with higher prevalence of IPV could have higher mental health risks, making it important to develop site-specific strategies (Kundu et al., 2024; Saba et al., 2024).

The identified pattern of the south-central concentration of IPV and mental health burden is probably due to a complex of social, economic and cultural factors, such as gender norms, poverty and access to health services (Roth et al., 2020). The problem of urban and rural disparities implies that females in the city have a higher level of awareness and reporting ability, and females in the country might face underreporting as a result of stigmatization (Gunarathne et al., 2024; Kurvinen et al., 2025). Household internet access and material resources suggest potential for digital and community-based interventions to screen and educate women (Meekers et al., 2013; Miah & Ullah, 2025a.

Therefore, these findings are consistent with studies in Nepal, Bangladesh, Canada and Mozambique; emotional and physical IPVs in Zambia are strongly associated with anxiety and depression, with the highest burden concentrated in southern-central provinces (Zhang et al., 2019; Miao et al., 2024; Rahman et al., 2024; Antabe et al., 2025; Barini et al., 2025; Shaikh, 2025; Miah and Kabir, 2026).

### Policy implications

The findings demonstrate that policy-based integrated mental health and IPV interventions are urgently needed in Zambia, with certain implications on policies: (i) Geographically focused interventions: Focus on mental health screening and IPV prevention at high-burden provinces (Copperbelt, Central, Southern and Eastern). (ii) Integrated service provision: Integrate primary healthcare and mental health care with IPV support, such as legal protection, psychosocial counseling and safe shelter. (iii) Digital and community-based interventions: Use the internet and media to create awareness, offer counseling and lower the stigma of IPV and mental health. (iv) SDG-congruent interventions: Position interventions in line with SDG 3 (Good Health and Well-being), SDG 5 (Gender Equality) and SDG 10 (Reduced Inequalities) to have sustainable effects. Overall, policies should target high-burden provinces, integrating mental health and IPV services through community and digital platforms.

### Strengths

The study was the first national representative research in Zambia to integrate mental health measures using the PHQ-9 and GAD-7 within the ZDHS, capturing data from a large sample of 13,951 women. The inclusion of these validated instruments allows robust assessment of depression and anxiety at a population level. The study applied rigorous conceptual and statistical processes for model selection, enhancing the reliability of the findings. Additionally, spatial mapping techniques enable the identification of provincial hotspots, informing targeted interventions for intimate partner violence (IPV) and mental health. The strong survey design, including appropriate weighting and stratification, ensures generalizability of results to Zambian women aged 15–49 years.

### Limitations

It was a cross-sectional design; therefore, it was not possible to draw a causal inference. Additionally, it was based on self-reported data to drive stigma or recall bias. Besides, residual confounding existed. Future studies should use longitudinal designs to better assess causality. Mixed-methods approaches and validated objective measures may reduce reporting bias. Including additional contextual and behavioral factors could help minimize residual confounding and improve study robustness.

### Theoretical contributions

This study is informed by an ecological stress trauma framework and conceptualizes IPV as a multilevel psychosocial stressor that outlines women’s mental health outcomes and their spatial inequalities across Zambia.

## Conclusions

This article generates strong, nationally representative data that indicate that physical, emotional and general IPV are strongly linked with depression and anxiety in Zambian women. The spatial distribution gives emphasis on southern-central provinces as high-priority areas that need intervention. These results underpin the idea of poor-law-tailored, SDG-consistent and culturally mindful interventions based on mental health services and IPV prevention and response to enhance the health and well-being of women in Zambia. These findings suggest the urgent need for targeted maternal health policies and interventions to address IPV, supporting progress toward SDG 3 (Good Health and Well-being) and SDG 5 (Gender Equality). Spatially informed strategies can help prioritize high-burden regions for effective resource allocation and prevention programs.

These findings are likely generalizable to other low- and middle-income countries (LMICs), where comparable socioeconomic settings, gender control inequities and inadequate access to mental health services similarly improve women’s vulnerability to intimate partner violence and common mental disorders. The co-occurrence of IPV and depression–anxiety observed in Zambia reflects structural and cultural shapes widely recognized across LMIC settings, signifying that the acknowledged relations are not context-specific but revealing of broader provincial and global trends.

## Supporting information

10.1017/gmh.2026.10261.sm001 Miah and Uddin supplementary material Miah and Uddin supplementary material

## Data Availability

This secondary dataset is available on request from the DHS program website at: (https://www.dhsprogram.com/data).

## Abbreviation

aOR: adjusted odds ratio
CI: confidence interval
DHS: Demographic and Health Survey
GAD-7: Generalized Anxiety Disorder-7
GBV: gender-based violence
IPV: intimate partner violence
LMICs: lower middle-income countries
LRT: likelihood ratio test
PHQ-9: Patient Health Questionnaire-9
PTSD: post-traumatic stress disorder
RECMTH: Mental Health and Well-being Module
SDG: Sustainable Development Goal
SSA: Sub-Saharan Africa
STROBE: Strengthening the Reporting of Observational Studies in Epidemiology
USAID: United States Agency for International Development
VIF: variance inflation factor
WHO: World Health Organization
YLDs: years lived with disability
ZDHS: Zambia Demographic and Health Survey

## Supplementary material

The supplementary material for this article can be found at http://doi.org/10.1017/gmh.2026.10261.

## Long descriptions

### Table 1. Long description

The table consists of five columns and four rows of data.

* Row 1: Physical I P V. Defined as physical aggression by a partner. Examples include being pushed, shaken, slapped, punched, kicked, strangled, attacked with a weapon, or having hair pulled. D H S codes are d 1 0 5 a, d 1 0 5 b, d 1 0 5 c, d 1 0 5 d, d 1 0 5 e, d 1 0 5 f, and d 1 0 5 j. Coding is 1 if any are experienced, otherwise 0.

* Row 2: Emotional I P V. Defined as emotional harm or fear. Examples include humiliation, threats, and insults. D H S codes are d 1 0 3 a, d 1 0 3 b, and d 1 0 3 c. Coding is 1 if any are experienced, otherwise 0.

* Row 3: Sexual I P V. Defined as a sexual act forced by a partner. Examples include forced intercourse or unwanted sexual acts. D H S codes are d 1 0 5 h, d 1 0 5 i, and d 1 0 5 k. Coding is 1 if any are experienced, otherwise 0.

* Row 4: Any I P V (composite). Defined as exposure to at least one type of I P V. Examples include physical, emotional, or sexual I P V. Variables include all physical, emotional, and sexual codes listed above. Coding is 1 if any I P V is experienced, otherwise 0.

Navigate back to Table 1..

### Table 2. Long description

The table presents data for 13,951 women, categorized by Depression (P H Q 9 score greater than or equal to 10) and Anxiety (G A D 7 score greater than or equal to 10). Each outcome is split into No and Yes columns with associated p-values.

I P V exposures section:

* Physical I P V: Yes (7.73 percent depression, 5.36 percent anxiety); p-values are less than 0.001 and 0.009 respectively.

* Emotional I P V: Yes (9.66 percent depression, 7.14 percent anxiety); p-values are less than 0.001 and 0.002 respectively.

* Sexual I P V: Yes (1.93 percent depression, 2.98 percent anxiety); p-values are 0.212 and 0.014 respectively.

* Any I P V: Yes (11.59 percent depression, 10.71 percent anxiety); p-values are both less than 0.001.

Sociodemographic characteristics section (selected significant findings):

* Maternal age: Depression and anxiety rates increase with age, peaking at 2.00 percent and 1.57 percent respectively for the 35 to 49 age group (p-values 0.001 and less than 0.001).

* Maternal education: Secondary or higher education shows higher depression rates (1.74 percent) compared to no education (1.25 percent), with a p-value of 0.041.

* Number of unions: More than once (2.40 percent depression) vs once (1.39 percent), p-value 0.004.

* Residence type: Urban areas show higher rates (1.76 percent depression, 1.48 percent anxiety) than rural areas (1.27 percent depression, 0.98 percent anxiety), with p-values of 0.016 and 0.007.

* Internet use: Users have higher rates (2.12 percent depression, 1.70 percent anxiety) than non-users (1.27 percent depression, 1.04 percent anxiety), with p-values less than 0.001 and 0.002.

Navigate back to Table 2..

### Table 3. Long description

The table presents data for a sample size N = 13, 951. It is divided into two main outcome sections: Anxiety and Depression. Columns include Outcome, Exposure, Unadjusted O R (95% C I) with P-value, and Adjusted O R (95% C I) with P-value.

For Anxiety:

* Physical I P V (Yes): Unadjusted O R 1.84 (0.91–3.69), P = 0.085; Adjusted O R 1.84 (0.89–3.83), P = 1.84.

* Sexual I P V (Yes): Unadjusted O R 2.50 (0.95–6.59), P = 0.065; Adjusted O R 2.46 (0.90–6.70), P = 0.077.

* Emotional I P V (Yes): Unadjusted O R 2.11 (1.15–3.89), P = 0.015; Adjusted O R 1.97 (1.05–3.72), P = 0.034.

* Any I P V (Yes): Unadjusted O R 2.06 (1.23–3.44), P = 0.005; Adjusted O R 2.05 (1.20–3.50), P = 0.008.

For Depression:

* Physical I P V (Yes): Unadjusted O R 3.32 (1.93–5.69), P < 0.001; Adjusted O R 3.89 (2.16–6.95), P < 0.001.

* Sexual I P V (Yes): Unadjusted O R 1.64 (0.58–4.67), P = 0.53; Adjusted O R 1.75 (0.60–5.06), P = 0.298.

* Emotional I P V (Yes): Unadjusted O R 3.29 (2.01–5.40), P < 0.001; Adjusted O R 3.50 (2.09–5.86), P < 0.001.

* Any I P V (Yes): Unadjusted O R 2.59 (1.64–4.07), P < 0.001; Adjusted O R 2.90 (1.80–4.67), P < 0.001.

Reference category for all exposures is No. Adjusted models account for maternal age, education, wealth, residence, and other demographic factors.

Navigate back to Table 3..

### Figure 1. Long description

A geographical map of Zambia divided into ten provinces. A vertical color scale on the right, titled Anxiety percent, ranges from light blue at 2 percent to dark blue at 4 percent. A north arrow is in the top left and a 400 kilometer scale bar is at the bottom left.

Regional distribution of anxiety:

* Northern regions: Northern, Luapula, and Muchinga provinces are light blue, indicating lower anxiety levels near 2 percent.

* Central and Eastern regions: Central and Eastern provinces are medium blue, approximately 3 percent. Copperbelt province is the darkest blue, representing the highest anxiety level at 4 percent.

* Southern and Western regions: Western province is a solid medium-dark blue. Southern and Lusaka provinces are medium blue, similar to the Central region. North-Western province is light blue, matching the northern provinces.

Navigate back to Figure 1..

### Figure 2. Long description

A geographical map of Zambia is divided into ten provinces, each shaded according to a color scale representing Depression percentage from 2 percent (light cream) to 4 percent (dark orange).

* In the North, Northern and Luapula provinces are shaded light orange, while Muchinga is light cream.

* In the West and Northwest, Western province is medium-dark orange, while North-Western is light cream.

* In the Center, Copperbelt province shows the highest concentration with the darkest orange shading, followed by Central province in medium orange.

* In the East, Eastern province is shaded medium orange.

* In the South, Southern and Lusaka provinces are shaded medium orange.

The map includes a North arrow in the top-left corner, a 400 kilometer scale bar at the bottom-left, and axes labeled Latitude and Longitude. A vertical legend on the right titled Depression percent provides the color gradient with markers at 2 percent, 2.5 percent, 3 percent, 3.5 percent, and 4 percent.

Navigate back to Figure 2..

### Figure 3. Long description

A geographical map of Zambia divided into ten provinces, colored by I P V percentage. A legend on the right indicates a color gradient from light pink (2 percent) to dark burgundy (4 percent).

* In the North, Northern and Luapula provinces are medium pink, while Muchinga in the Northeast is the lightest shade at approximately 2 percent.

* The Central and Southern provinces are the darkest burgundy, representing the highest prevalence near 4 percent.

* Eastern province is a deep pink, approximately 3.5 percent.

* Lusaka province in the Southeast is light pink, around 2.5 percent.

* To the West, North-Western, Copperbelt, and Western provinces are shaded in medium-light pink, ranging between 2.5 percent and 3 percent.

A north arrow is located in the top-left corner, and a 400 kilometer scale bar is positioned at the bottom-left.

Navigate back to Figure 3..
