# Supplementary figures and images for: Intimate partner violence and the dual burden of anxiety and depression among women in Zambia: Spatial inequalities and implications for the sustainable development goals
Source: Glob Ment Health (Camb). 2026 Jun 25;13:e143. doi: 10.1017/gmh.2026.10261 (PMC13373271; doi:10.1017/gmh.2026.10261)

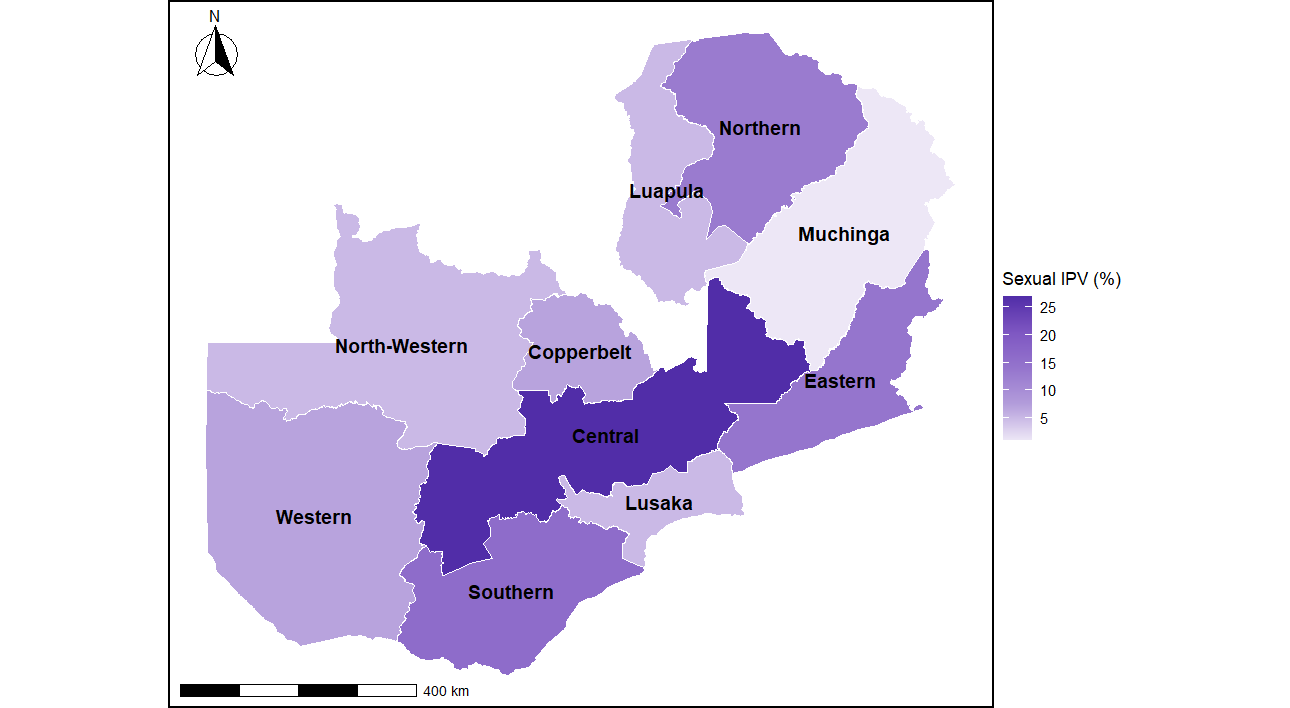

Supplement: Miah and Uddin supplementary material [file S2054425126102611sup001.zip › Supplementary Figure S5.tiff]

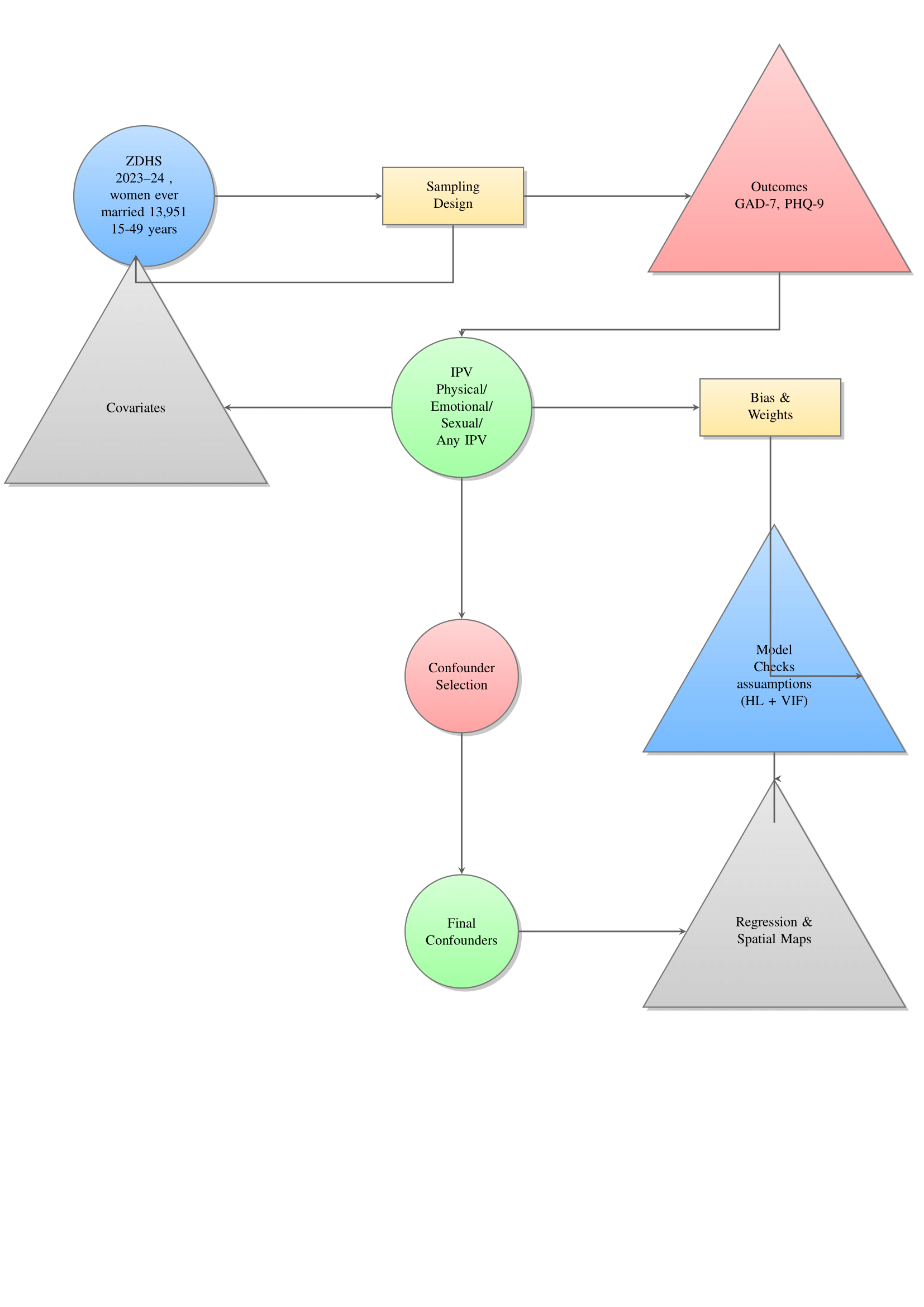

Supplement: Miah and Uddin supplementary material [file S2054425126102611sup001.zip › Supplementary Figure S1.png]

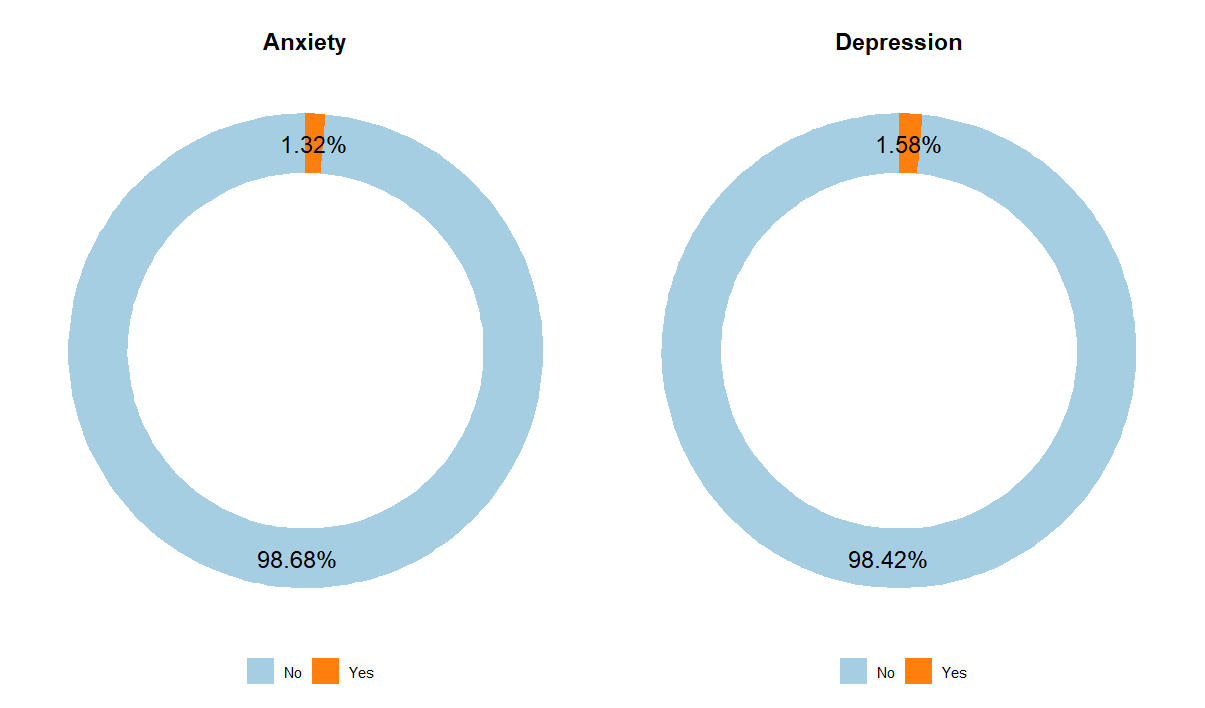

Supplement: Miah and Uddin supplementary material [file S2054425126102611sup001.zip › Supplementary Figure S2.tiff]

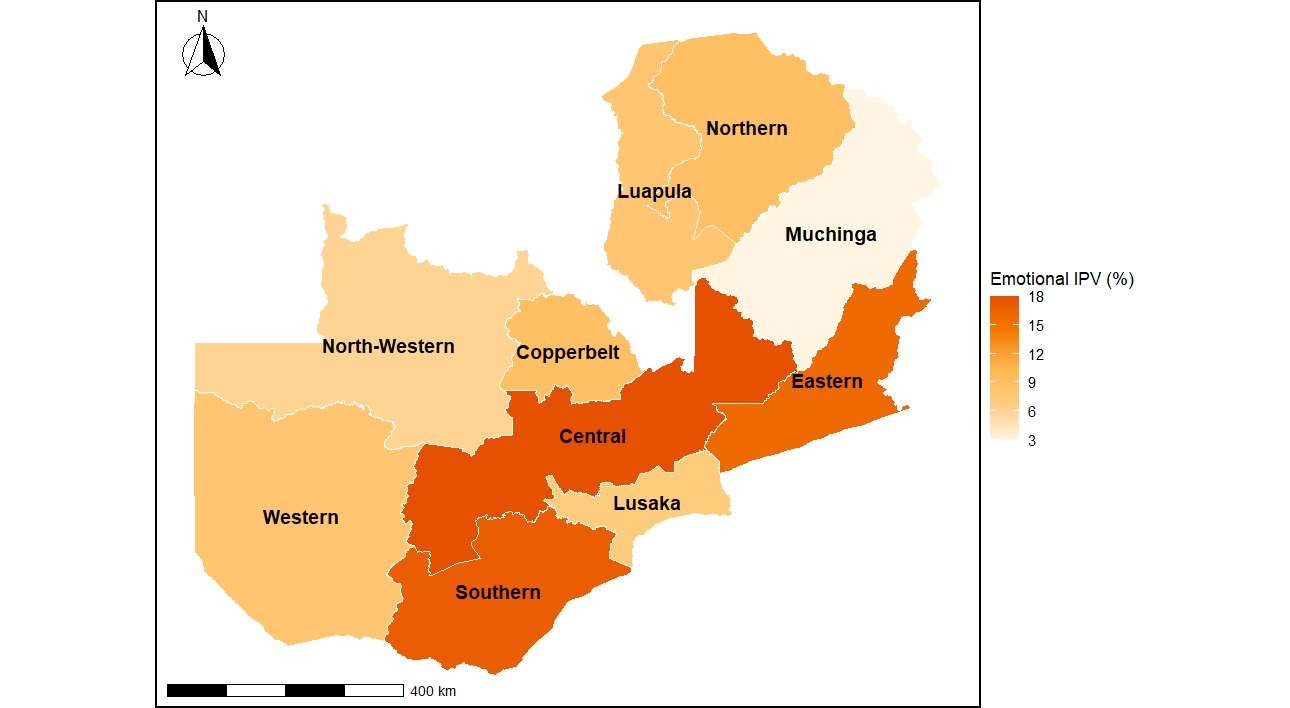

Supplement: Miah and Uddin supplementary material [file S2054425126102611sup001.zip › Supplementary Figure S3.tiff]

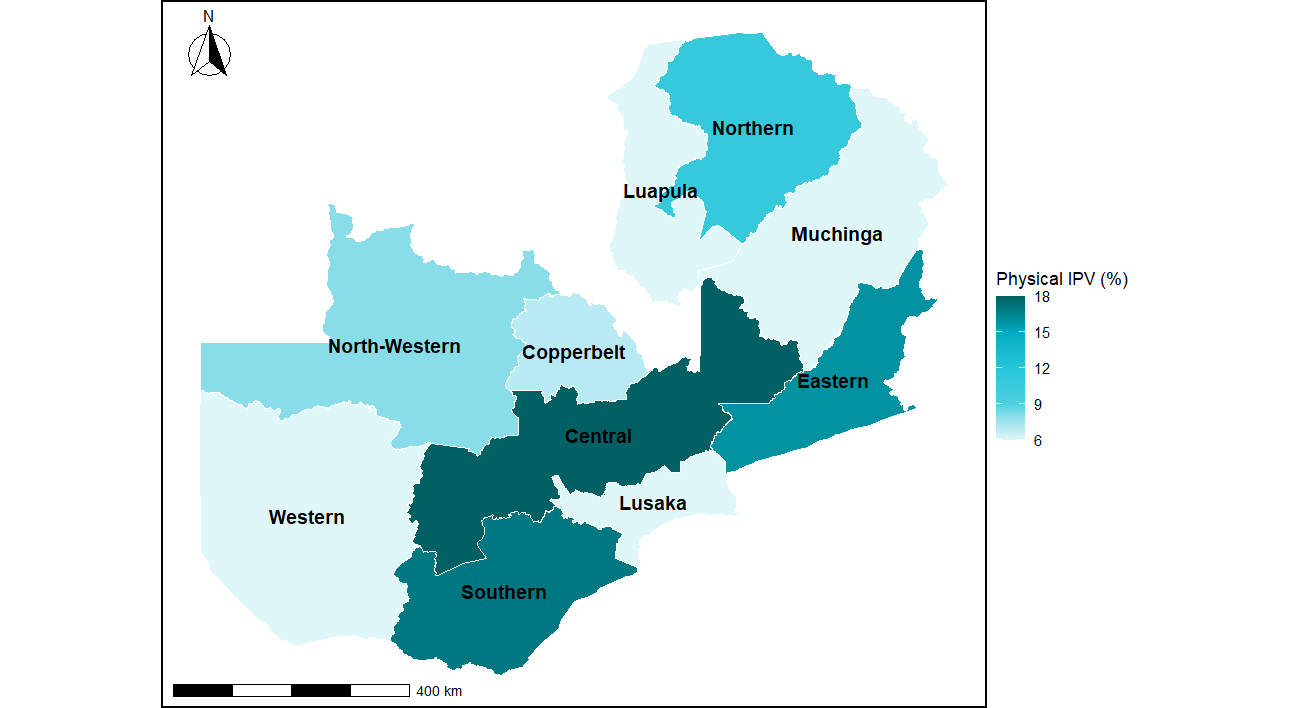

Supplement: Miah and Uddin supplementary material [file S2054425126102611sup001.zip › Supplementary Figure S4.tiff]
