# Supplementary material for: Intimate partner violence and the dual burden of anxiety and depression among women in Zambia: Spatial inequalities and implications for the sustainable development goals
Source: Glob Ment Health (Camb). 2026 Jun 25;13:e143. doi: 10.1017/gmh.2026.10261 (PMC13373271; doi:10.1017/gmh.2026.10261)
Supplement: Peer review history

# Reviewer report: Review: Intimate partner violence and the dual burden of anxiety and depression among women in Zambia: Spatial inequalities and implications for the sustainable development goals — R0/PR2

**DOI:** 10.1017/gmh.2026.10261.pr2

**Authors:** Zacharie Tsala Dimbuene

**Affiliations:** Department of Population Sciences , Universite de Kinshasa, Congo (the Democratic Republic of the)

Title: Intimate Partner Violence and the Dual Burden of Anxiety and Depression Among Women in Zambia: Spatial Inequalities and Implications for the Sustainable Development Goals

The paper addresses an important issue by linking intimate partner violence (IPV) and mental health which are both critical health challenges in developed and developing countries. Overall, the paper is intelligible but shows a bit of neglect during the preparation of the manuscript. Here is the breakdown of my comments: 

1. References are incomplete (e.g., many references are n.d.) and other references are mispresented 

2. Table 2. Percentages have been calculated in columns. It is not clear what this means and how this was truly interpreted. It makes more sense to calculate row percentages and that might be interpreted as rate; and this can be compared among the NO (no IPV/components) and YES groups. See Table 2. This is critical to better understand and interpret the findings. 

3. Maps about anxiety and depression and the Figure about anxiety and depression (ln 303) seem to contradict. In Fig less than 2% of ever-married women experienced anxiety and/or depression. In the maps, I can see that the percentage of women with anxiety and depression ranged from 0 to >30% (depression), and from 0 to >20% (anxiety). What is the explanation because the simple logic does not hold. 

4. Pie chats with binary variables are irrelevant and you know the “why”

5. Check Table 1 (e.g. burnedd105f: actsd105k:)

6. ln 263 province(division)

7. ln 295 - Among 13, 951 ever married women

Decision: Major Revision

# Reviewer report: Review: Intimate partner violence and the dual burden of anxiety and depression among women in Zambia: Spatial inequalities and implications for the sustainable development goals — R0/PR3

**DOI:** 10.1017/gmh.2026.10261.pr3

Abstract

Authors should correct grammar in the second last sentence “These findings highlight to provide evidence based, actionable insights for policymakers to integrated mental health and IPV prevention in high burden province to meet the target of SDG 3 and 5”.

Line 5: On background information. There is a problem with word choice. ‘Intimate Partner Violence (IPV) highlights an important global public health issue….”

The entire background information paragraph should be rephrased; it lacks clarity and several grammar issues are evident. For example, the authors should simply say ‘IPV is an important global health issue….’

‘….IPV is barrier to achieving….’

Objective lines 9, 10: authors to address grammar issues.

Method lines 12-18: Authors to address glaring grammar issues. There is also lack of clarity.

Impact statement lines 34-54: This section is poorly written with several grammatical errors.

Introduction lines 34-54: Grammar issues. Issue on citation on lines 60, 67, and 81.

On lines 133-135: Assess the precise associations between all dimensions of IPV (physical, sexual, emotional, and any IPV) and mental health indicators (anxiety and depression) among women aged 15-49 years in Zambia using the 2024 ZDHS data. How did authors assess precise association? Statistically we estimate. Moreover, there is no such a thing as precise estimate!

Methodology

Line 163, 177, and 196: errors citation

Variables (lines 183-196)

Authors should address glaring grammar issues. Paragraph on lines 183-196 is extremely poorly written.

Authors should explain how co-occurring symptoms of the two disorders were handled in the analysis. How respondent who were on treatment for either disorder but their score were was below threshold handled?

Exposure variables.

Does exposure to physical, emotional, or sexual IPV preclude exposure to the other forms? For example, does exposure to physical IPV preclude exposure to sexual or emotional IPV? This should be clear.

Choice of confounders

Authors claim to have selected confounders based on availability and relevant studies without citing any source.

Set of confounders in final model

Authors should justify why the final model included household wealth status alongside other factors such household ownership, household materials, household assets, from which is derived. Lines 259-264.

Results

Authors should rewrite this section. For example in lines 303-306, ‘Among all types of IPV prevalence, physical IPV showed about 2.3%, sexual IPV 1.1%, emotional IPV 3.15%, any kinds of IPV 4.5%’. This lacks clarity.

The Discussion, policy Implications, and strengths sections also need to be revised to ensure clarity and consistency with the previous sections

# Editor report: Recommendation: Intimate partner violence and the dual burden of anxiety and depression among women in Zambia: Spatial inequalities and implications for the sustainable development goals — R0/PR4

**DOI:** 10.1017/gmh.2026.10261.pr4

**Affiliations:** Fudan University, China

No accompanying comment.

# Editor report: Decision: Intimate partner violence and the dual burden of anxiety and depression among women in Zambia: Spatial inequalities and implications for the sustainable development goals — R0/PR5

**DOI:** 10.1017/gmh.2026.10261.pr5

**Affiliations:** London School of Hygiene & Tropical Medicine, United Kingdom of Great Britain and Northern Ireland

No accompanying comment.

# Reviewer report: Review: Intimate partner violence and the dual burden of anxiety and depression among women in Zambia: Spatial inequalities and implications for the sustainable development goals — R1/PR7

**DOI:** 10.1017/gmh.2026.10261.pr7

**Authors:** Zacharie Tsala Dimbuene

**Affiliations:** Department of Population Sciences , Universite de Kinshasa, Congo (the Democratic Republic of the)

I commend the authors did efforts to incorporate reviewers’ comments with gallantry. That has significantly improved the manuscript. However, I am not convinced by authors’ responses on the following. On page 40 (Map shows province-level prevalence of depression among ever-married women. National prevalence is low (about 2–4%), and the map highlights relative geographic variation rather than the national average.). If the national estimate of depression ranges (this is false because a national estimate is a point estimate), in no ways provincial estimates with range between 0 and 30%. Same can be said on anxiety.

Second, Figures reported in Table 2, especially between sociodemographics and (i) depression and (ii) anxiety are totally wrong. Let’s take an example. For youth aged 15-24, here are the Figures reported:

Depression:

Age group No Yes

15-24 5826 (42.39) 63 (30.43)

Just to take this example, I don’t know where this Figures come. First, the sum should be 100%. This is not the case. Correct maths should be depression rate = 63/(5826+63)*100 = 1.07%. Therefore, 1.07% of youth suffered from depression vs. 98.93% who did not suffer from depression; etc.

With these two crucial comments which invalidate many statistics reported in the paper, I leave the Editors for final decision.

# Reviewer report: Review: Intimate partner violence and the dual burden of anxiety and depression among women in Zambia: Spatial inequalities and implications for the sustainable development goals — R1/PR8

**DOI:** 10.1017/gmh.2026.10261.pr8

None

# Editor report: Recommendation: Intimate partner violence and the dual burden of anxiety and depression among women in Zambia: Spatial inequalities and implications for the sustainable development goals — R1/PR9

**DOI:** 10.1017/gmh.2026.10261.pr9

**Affiliations:** Fudan University, China

The current presentation of data in Table 2 and the Figures lacks consistency, which likely underlies Reviewer 1’s confusion. Specifically, Table 2 switches between column percentages for sociodemographic characteristics and row percentages for IPV exposures. Furthermore, the shift from reporting general proportions in Figures 1 and 2 to prevalence rates in Figure 3 creates an inconsistent narrative. To ensure clarity, the authors should standardize the presentation—ideally using row percentages throughout Table 2 to consistently show the distribution of outcomes across groups—and explicitly define the metrics used in each Figure caption.

# Editor report: Decision: Intimate partner violence and the dual burden of anxiety and depression among women in Zambia: Spatial inequalities and implications for the sustainable development goals — R1/PR10

**DOI:** 10.1017/gmh.2026.10261.pr10

**Affiliations:** London School of Hygiene & Tropical Medicine, United Kingdom of Great Britain and Northern Ireland

No accompanying comment.

# Editor report: Recommendation: Intimate partner violence and the dual burden of anxiety and depression among women in Zambia: Spatial inequalities and implications for the sustainable development goals — R2/PR12

**DOI:** 10.1017/gmh.2026.10261.pr12

**Affiliations:** Fudan University, China

The presentation of row and column percentages in Table 3 is still inconsistent. Currently, rows under “IPV exposures” are reported as column percentages, whereas rows under “Sociodemographic Characteristics” use row percentages. I recommend standardizing the table by also presenting the values for “IPV exposures” as row percentages.

# Editor report: Decision: Intimate partner violence and the dual burden of anxiety and depression among women in Zambia: Spatial inequalities and implications for the sustainable development goals — R2/PR13

**DOI:** 10.1017/gmh.2026.10261.pr13

**Affiliations:** London School of Hygiene & Tropical Medicine, United Kingdom of Great Britain and Northern Ireland

No accompanying comment.

# Editor report: Recommendation: Intimate partner violence and the dual burden of anxiety and depression among women in Zambia: Spatial inequalities and implications for the sustainable development goals — R3/PR15

**DOI:** 10.1017/gmh.2026.10261.pr15

**Affiliations:** Fudan University, China

No accompanying comment.

# Editor report: Decision: Intimate partner violence and the dual burden of anxiety and depression among women in Zambia: Spatial inequalities and implications for the sustainable development goals — R3/PR16

**DOI:** 10.1017/gmh.2026.10261.pr16

**Affiliations:** London School of Hygiene & Tropical Medicine, United Kingdom of Great Britain and Northern Ireland

No accompanying comment.
